# Geopolymer-Based Stabilization of Heavy Metals, the Role of Chemical Agents in Encapsulation and Adsorption: Review

**DOI:** 10.3390/polym17050670

**Published:** 2025-03-01

**Authors:** Francesco Genua, Isabella Lancellotti, Cristina Leonelli

**Affiliations:** Department of Engineering ‘Enzo Ferrari’, University of Modena and Reggio Emilia, Via Vivarelli 10, 41125 Modena, Italy; francesco.genua@unimore.it (F.G.); cristina.leonelli@unimore.it (C.L.)

**Keywords:** geopolymer, heavy metal, chemical agents, encapsulation

## Abstract

This review provides a comprehensive analysis of the role of chemical agents in enhancing the performance of geopolymers for the stabilization and adsorption of heavy metals. Geopolymers, synthesized from aluminosilicate sources activated under alkaline conditions, are recognized for their versatile structural and environmental benefits, including low carbon emissions and high chemical resistance. Their unique Si-O-Al framework supports both stabilization/solidification (S/S) and adsorption processes, making them an ideal polymeric matrix for the immobilization of hazardous heavy metals in contaminated environments. The review categorizes the heavy metal immobilization mechanisms into physical encapsulation, ion exchange, hydroxide precipitation, and chemical complexation, depending on the specific metal species and geopolymer formulation. The introduction of chemical stabilizing agents, such as dithiocarbamate, sodium sulfide, and trimercaptotriazine, significantly improves the encapsulation efficacy of geopolymers by promoting targeted reactions and stable metal complexes. These agents enable the effective S/S of metals, such as lead, cadmium, and chromium, reducing their leachability and environmental impact. In addition to solid waste management applications, geopolymers have shown promising adsorption capabilities for aqueous contaminants, with chemical modifications further increasing their affinity for specific heavy metals. This review evaluates the impact of different agents and synthesis conditions on the overall performance of geopolymers in heavy metal immobilization, highlighting advances in environmental applications and future research directions for sustainable hazardous waste treatment.

## 1. Development of Geopolymers

Geopolymers are a class of inorganic polymers, mainly composed of aluminosilicate minerals, which have attracted attention for their potential as environmentally friendly alternatives to traditional materials, such as Portland cement. The concept of geopolymers was first proposed by Joseph Davidovits in the 1970s while researching fire-resistant materials, leading to the term ’geopolymer’ to describe materials formed through the alkaline activation of aluminosilicate sources [1]. In particular, Davidovits’ work was driven by the desire to create building materials with a lower carbon footprint and improved durability compared to conventional clinker-based cement [2].

The earliest studies of geopolymer-like reactions date back to the 1930s, with early work investigating the role of alkaline activators in the transformation and consolidation of aluminosilicate precursors at room temperature. However, it was not until the energy crises of the 1970s that interest in alternative binders, such as geopolymers, surged as researchers sought to reduce the environmental impact of construction materials [3]. Since then, geopolymers have been recognized for their low carbon emissions, high compressive strength, chemical resistance, and durability due to their unique three-dimensional network structure [4].

Research into geopolymers has expanded significantly since the 1990s, with studies focusing on the composition, properties, and potential applications of these materials in various fields, including waste treatment, fireproofing, and high-performance concrete [5]. This increased interest in geopolymers is due to their adaptability in using industrial by-products, such as fly ash and slag, as feedstocks, contributing to waste reduction and sustainable construction practices [6,7]. In addition, ongoing research has demonstrated the effectiveness of geopolymers in immobilizing heavy metals, offering promising solutions for pollution control [8,9,10].

As a result, geopolymers are increasingly being considered as sustainable materials with a wide range of applications, from conventional construction to specialized environmental remediation projects [7,11,12].

In the last few decades, geopolymers have found roles beyond conventional construction, including in the stabilization and immobilization of hazardous materials, such as heavy metals and radioactive waste [13,14]. Researchers have recognized the geopolymer matrix as a robust framework for containing contaminants, significantly reducing leachability and providing a stable solution for waste disposal [15]. This progress highlights the potential of geopolymers not only as sustainable building materials but also as strategic environmental solutions [5].

### 1.1. Reaction Mechanism of Geopolymers

**Figure 1:**
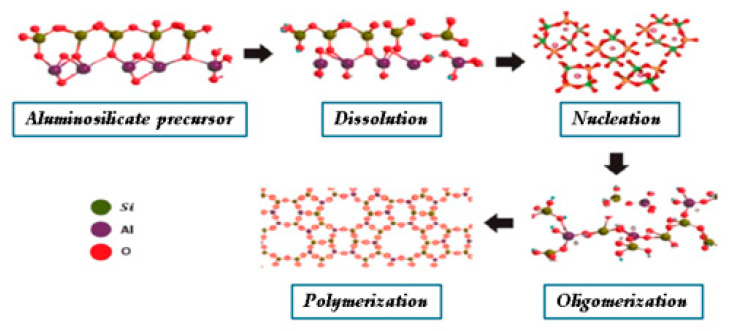
Reactions involved in geopolymerization and four stages of geopolymerization: dissolution, nucleation, oligomerization, and polymerization reprinted with permission from [26] Copyright 2017, Springer, Cham.

The geopolymerization process is a complex chemical reaction that transforms aluminosilicate sources into a stable, cross-linked, three-dimensional network. The process generally involves four stages as shown in Figure 1: dissolution, where the aluminosilicate particles break down in an alkaline solution; nucleation, where initial molecular clusters are formed; oligomerization, where small chains and rings are formed; and finally, polymerization, where the solid matrix is formed [16,17]. The process is controlled by the concentration of the alkaline activator, Si/Al ratio, curing temperature, and additives, which together influence the rate and quality of geopolymerization [18,19].

During dissolution, reactive aluminosilicate species, like SiO_4_^4−^ and AlO_4_^5−^ tetrahedra, are released and combine with cations (e.g., Na⁺ or K⁺) that neutralize the negative charges [20,21]. In the subsequent nucleation and polymerization stages, these species form a stable structure of Si-O-Si and Si-O-Al covalent bonds through a condensation reaction, resulting in a rigid inorganic matrix with zeolite-like properties [22]. This matrix is uniquely suited to encapsulate various types of contaminants due to its structural integrity and the presence of negative charges that attract positively charged ions, such as heavy metals. Thus, geopolymerization can effectively immobilize contaminants by chemical bonding or physical encapsulation within the matrix, which is critical for environmental applications [23].

Studies on the geopolymerization of various aluminosilicate precursors, including metakaolin, blast-furnace slag, and industrial by-products, show that by adjusting the reaction conditions (e.g., alkali concentration and curing environment), the mechanical and chemical properties of the material can be tailored for specific applications, from high-strength concrete to hazardous waste containment [24,25].

### 1.2. Properties of Geopolymers

Geopolymers have unique properties that distinguish them from other inorganic polymers, i.e., conventional cementitious materials, including high compressive strength, durability, and resistance to chemical and thermal degradation [27]. These materials are not only able to withstand harsh environmental conditions but also provide the superior immobilization of hazardous substances due to their molecular structure [23]:

- Mechanical Properties: Geopolymers exhibit high compressive strength, often comparable to or exceeding that of traditional Portland cement, with values ranging from 20 MPa to over 100 MPa depending on the precursor materials and activation conditions [18,28]. This mechanical robustness, combined with excellent flexural and tensile properties, makes them suitable for structural applications, particularly in aggressive environments where traditional binders may fail.
- Chemical and Thermal Resistance: Due to their low calcium content and cross-linked molecular structure, geopolymers are highly resistant to sulfate, acid, and chloride attacks, as well as to high temperatures. This property is particularly beneficial in scenarios where materials are exposed to corrosive agents or extreme heat, such as in fire-resistant structures or chemical waste containment [29,30,31]. Geopolymers have demonstrated stability at temperatures exceeding 1000 °C, making them ideal for fireproofing and refractory applications [32].
- Environmental performance: The ability of geopolymers to incorporate industrial by-products, such as fly ash and slag, reduces the environmental impact associated with waste disposal and raw material extraction [4,7]. This property, combined with their lower carbon footprint compared to Portland cement, positions geopolymers as an environmentally friendly alternative in construction and waste management. In addition, their high immobilization efficiency for heavy metals and other contaminants enables them to play an important role in environmental remediation.
- Applications in Waste Stabilization: Geopolymers’ unique properties make them highly effective in the stabilization and solidification of hazardous wastes, such as heavy metals (e.g., Pb, Zn, Cd) and radioactive elements (e.g., Cs, Sr). Studies have shown that geopolymers immobilize these substances through physical encapsulation and chemical bonding, resulting in materials that resist leaching under various environmental conditions [13,14,22]. Geopolymers also exhibit strong adsorption capacities for heavy metals, increasing their effectiveness in immobilizing contaminants [33,34,35].

## 2. Introduction to Heavy Metal Immobilization in Geopolymers

Heavy metal contamination has become a significant environmental issue in various regions across the globe, primarily due to human activities, such as mining, smelting, and coal combustion, largely as a result from the improper disposal of the wastes generated by these activities [36]. Unlike organic pollutants, heavy metals are non-biodegradable and can persist in the environment, forming stable and irreversible compounds. These metals can exist in different chemical forms, making their environmental impact more complex. Heavy metals are generally categorized into cationic and anionic types, both of which pose serious environmental risks. Heavy metals, such as lead (Pb), cadmium (Cd), chromium (Cr), nickel (Ni), iron (Fe), and zinc (Zn), pose significant environmental and health risks due to their toxicity and persistence in ecosystems. Geopolymers, as aluminosilicate-based matrices with unique structural properties, have been extensively studied for their potential to immobilize these hazardous elements [37,38,39]. The immobilization of heavy metals within the geopolymer matrix is driven by both physical and chemical mechanisms that reduce leachability and increase stability under different environmental conditions [40]. Due to their three-dimensional framework and the negative charge of alumina tetrahedra, geopolymers provide an ideal matrix for the capture of positively charged metal ions, making them effective in the treatment of wastes from industrial, municipal, and mining processes [41,42].

### 2.1. Stabilization Mechanism of Heavy Metals in Geopolymers

The immobilization of heavy metals in the geopolymer matrix occurs through a variety of chemical and physical interactions, which can be broadly classified into solidification/stabilization (S/S) (or encapsulation) and adsorption processes [9,40]. These mechanisms ensure the retention and encapsulation of metal ions within the matrix. While both S/S and adsorption serve to immobilize heavy metals, they differ in their approach and the strength of the metal binding. Solidification/stabilization (S/S) is a process in which contaminants, such as heavy metals, are physically and chemically encapsulated in a solid material to reduce their mobility and prevent their release into the environment. In this process, contaminants are either physically trapped or chemically bound within the solid matrix, limiting their ability to leach or diffuse. The aim of S/S is to render contaminants stable for long periods of time, thereby reducing the long-term risks of contamination [4,37].

Adsorption, on the other hand, is a physical process in which molecules or ions of a contaminant attach themselves to the surface of an adsorbent material, such as a geopolymer, without changing their chemical structure. This phenomenon relies on physical forces, such as electrostatic attraction or van der Waals forces. While adsorption is effective in removing contaminants from the environment, it does not provide long-term stabilization, such as S/S. Adsorption is generally reversible, meaning that contaminants can be released if the conditions change [4,37].

#### 2.1.1. Solidification/Stabilization in Geopolymers

**Figure 2:**
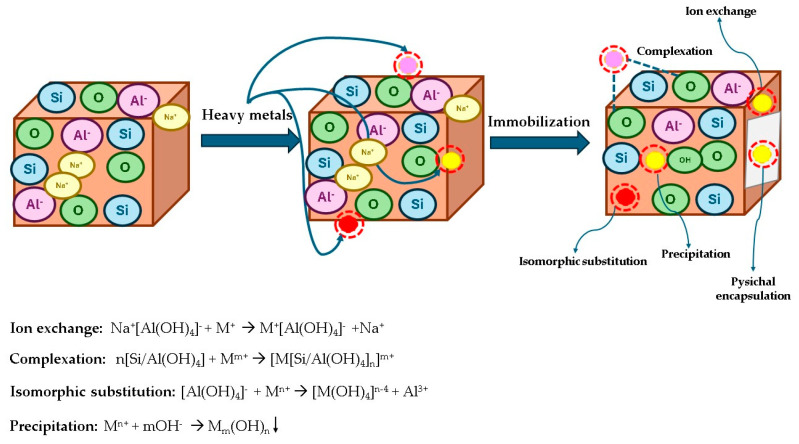
Different mechanism of stabilization of heavy metals.

This approach involves encapsulating metal ions within the geopolymer framework, reducing their solubility and potential release into the environment. Solidification refers to the process of incorporating hazardous wastes into a solid matrix to reduce their solubility, thereby minimizing the risk of leaching. Stabilization, on the other hand, alters the chemical state of the contaminants, making them less soluble and less prone to migration. In the case of geopolymers, these two processes occur simultaneously. The geopolymers, through their unique three-dimensional Si-O-Al framework, form a robust matrix that physically encapsulates the heavy metals while chemically binding them to prevent their release into the environment. Here are the mechanisms through which solidification/stabilization occurs (Figure 2):

- Physical encapsulation: The physical encapsulation mechanism in geopolymer matrices refers to the process by which heavy metal ions are immobilized within the geopolymer structure through physical confinement, rather than chemical bonding [41,42,43,44]. This mechanism involves the entrapment of metal ions within the three-dimensional framework of the geopolymer, which significantly reduces their mobility and leaching potential [41,43]. Due to their dense and compact structure, geopolymers create a barrier that restricts the movement of contaminants. The encapsulation process helps stabilize the metals, making them less likely to leach into the surrounding environment. Studies have shown that the high mechanical strength and low permeability of geopolymeric materials improve the efficiency of physical encapsulation, providing a stable matrix for hazardous materials [40,43]. The intrinsic microporosity of geopolymers provides an effective means of the physical encapsulation of heavy metals, preventing direct contact with leaching agents. An extensive analysis of the porosity characteristics of metakaolin-based geopolymer matrices has shown that their total porosity ranges from 30 to 55 vol%, with pore sizes between 0.01 and 0.54 μm. The specific surface area of these materials is reported to be between 15 and 100 m²/g, highlighting the significant microstructural variability influenced by synthesis parameters. This encapsulation mechanism effectively reduces metal mobility by forming a physical barrier that limits the release of ions [45].
- Precipitation: The precipitation mechanism of heavy metal stabilization in geopolymers is based on the chemical interaction between metal ions and the geopolymer matrix, where heavy metals are immobilized primarily by the formation of insoluble metal compounds, such as hydroxides, carbonates, phosphates, and other insoluble compounds due to the highly alkaline environment. This process plays a crucial role in enhancing the stability of the geopolymers by reducing the solubility and mobility of heavy metals within the matrix, thereby reducing their leaching potential under various environmental conditions [44,45].

During the geopolymerization process, the alkalinity of the activator helps to dissolve the metal cations present in the precursor material. These metal ions, when in contact with the geopolymeric gel, react with available anions (such as hydroxides or silicates) to form insoluble precipitates. These precipitates become embedded in the geopolymer network, effectively trapping the metal ions in a stable, solid form [42,43,45].

For example, lead (Pb) and chromium (Cr) have been observed to precipitate within the geopolymer matrix in the form of metal hydroxides, such as Pb(OH)_2_ and Cr(OH)_3_, which are known to be less soluble under neutral and alkaline conditions, thus enhancing the immobilization of these toxic metals [41,42]. The formation of these precipitates significantly reduces the leachability of metals from the geopolymer, thus ensuring the long-term stability of the treated material.

- Ion exchange: Charge balance plays a key role in the stabilization of heavy metals in geopolymers. In the geopolymer matrix, aluminium ions (Al³⁺) are tetrahedrally coordinated with oxygen in the aluminosilicate framework, creating a negative charge on the [AlO_4_]⁻ tetrahedra. To maintain charge neutrality, metal cations, such as sodium (Na⁺), potassium (K⁺), or heavy metals, such as Pb²⁺, Cd²⁺, and Cu²⁺, can be incorporated into the structure. These metal ions replace sodium or potassium cations in the geopolymer structure, thereby balancing the negative charge generated by the substitution of aluminium for silicon in the framework [39,42,43]. This incorporation of metal ions serves multiple purposes. The metal cations can either remain as part of the geopolymer structure through ion exchange or form chemical bonds, thereby stabilizing the metal within the 3D network. For example, Pb²⁺ ions are frequently exchanged with sodium or potassium and become part of the aluminosilicate framework. In addition, metals, such as Cd²⁺, are incorporated by balancing the negative charge of aluminium tetrahedra, contributing to the overall mechanical stability and reducing the leaching potential of the metals [40,45]. Thus, charge balancing is a crucial aspect of geopolymer-based stabilization processes, ensuring both the structural integrity of the geopolymer and the immobilization of heavy metals, ultimately reducing their environmental impact.
- Isomorphic substitution: Isomorphic substitution is a key mechanism in the stabilization of heavy metals in geopolymers, where metal ions replace specific elements in the geopolymer structure, such as aluminium (Al³⁺). In geopolymers, the tetrahedral Al³⁺ ions in the aluminosilicate framework can be replaced by metal cations, such as Pb²⁺, Cu²⁺, or Zn^2+^. This substitution results in a matrix that is highly resistant to heavy metal leaching, making the material suitable for hazardous waste stabilization [37,40,42].
- Coordination bonds: Coordination bonds play an important role in the stabilization of heavy metals within the geopolymer matrix. Some metal ions, such as copper (Cu²⁺) and zinc (Zn²⁺), can form coordination bonds with functional groups present in the geopolymer, in particular the silanol (-Si-OH) and aluminol (-Al-OH) groups. These coordination bonds occur when metal ions interact with the oxygen atoms of these hydroxyl groups, resulting in a stable metal–hydroxyl complex. This interaction helps to immobilize the metal ions within the geopolymer structure, thereby reducing their mobility and leaching potential under environmental conditions [40,43].

#### 2.1.2. Adsorption in Geopolymers

- Adsorption is a process by which pollutants, such as heavy metals, are captured on the surface of a material. Unlike stabilization/solidification, where metals are incorporated into the geopolymer matrix through chemical bonding or physical encapsulation, adsorption primarily involves the interaction of metal ions with the surface of the adsorbent. In geopolymers, metal ions are adsorbed onto the negatively charged sites on the aluminosilicate surface, typically facilitated by functional groups, such as silanol (-Si-OH) and aluminol (-Al-OH) [4,38,46,47].

Geopolymers have emerged as an effective and eco-friendly adsorbent for the removal of heavy metals from wastewater due to their large surface area, high porosity, and the presence of various functional groups capable of binding metal ions [35,46]. The high surface area of porous geopolymers (which typically ranges between 16.2 m²/g and 216 m²/g [35,48,49]) allows for increased contact with pollutants, providing more adsorption sites for heavy metals, such as Pb²⁺, Cu²⁺, Cd²⁺, and Zn²⁺ [33,47,48]. Furthermore, the tunable nature of geopolymers allows their surface properties to be adjusted through modifications, such as the incorporation of nanoparticles or surfactants, which further enhance their adsorption capacity [35].

Adsorption in geopolymers is governed by various factors, including the surface chemistry, porosity, and the type of metal ion being adsorbed. Studies have shown that geopolymers can efficiently adsorb heavy metals, with removal capacities of up to 118.6 mg/g for Pb²⁺ [48]. The adsorption process is typically governed by pseudo-second-order kinetics and Langmuir isotherms, indicating a homogeneous distribution of adsorption sites and monolayer adsorption [46,49].

Compared to solidification/stabilization, which focuses on the immobilization of metals within a matrix, adsorption provides a more dynamic process where the metals can potentially be desorbed or the adsorbent regenerated. However, the stability of the metal ions adsorbed on the geopolymer surface ensures that adsorption is an effective technique for the treatment of wastewater containing toxic metals, especially when the adsorption sites are abundant and well-distributed; another advantage of adsorption is that desorption allows for the recovery of adsorbed heavy metals, which can then be used for other purposes [8,35,38,47].

- Ion exchange: The ion exchange mechanism in geopolymers is an essential process for the adsorption of heavy metals. Geopolymers, composed of an aluminosilicate framework, possess a negatively charged surface due to the presence of tetrahedral aluminium (Al³⁺) units within the structure. These negative charges attract positively charged metal ions from the surrounding environment, such as lead (Pb²⁺), cadmium (Cd²⁺), and copper (Cu²⁺), allowing for efficient ion exchange. The ion exchange process involves the replacement of alkali metal ions (e.g., Na⁺ or K⁺) with the heavy metal ions, which are incorporated into the geopolymer structure and thus immobilized within the material [8,48,50].
- Surface adsorption and complexation: Surface adsorption and complexation are key mechanisms by which geopolymers effectively adsorb heavy metals from aqueous solutions. Geopolymers have a large surface area and significant porosity, providing numerous active sites for the adsorption of metal ions. These porous materials are able to adsorb heavy metals, such as nickel (Ni²⁺), by utilizing functional groups on their surface, including aluminol (-Al-OH) and silanol (-Si-OH) groups. These functional groups facilitate the formation of stable metal–ligand complexes, which further enhance the stability of the adsorbed metals and reduce their leachability. Furthermore, the interaction between metal ions and the hydroxyl or silanol groups in geopolymers leads to the formation of stable complexes, which not only increase the adsorption capacity but also significantly reduce the chances of metal ions leaching out of the material over time [35,48].

This interaction between metal ions and functional groups provides long-term environmental protection by inhibiting the release of the adsorbed metals into the surrounding environment [51].

- Physical adsorption: Physical adsorption, involving mechanisms, such as van der Waals forces, plays a significant role in the immobilization of heavy metals in geopolymers. Unlike chemisorption, which involves the formation of strong chemical bonds, physical adsorption relies on weaker forces that occur between metal ions and the surface of the geopolymer. Although less stable than chemisorption, physical adsorption contributes to the overall effectiveness of the adsorption process by increasing the number of interactions between the metal ions and the geopolymer surface, thus further limiting the mobility of the contaminants [50,51].

### 2.2. Considerations for Heavy Metal Immobilization in Geopolymers

The immobilization of heavy metals in geopolymers is a multifaceted challenge, mainly due to the complex chemical interactions between the metals and the geopolymer matrix. Geopolymers formed via the alkali activation of aluminosilicate materials are effective in stabilizing and immobilizing heavy metals within their solid structure. This process is particularly effective for cationic metals, such as lead (Pb²⁺), zinc (Zn²⁺), and nickel (Ni²⁺), which are typically found in positively charged oxidation states. The positively charged nature of these metals allows them to be effectively incorporated into the geopolymer matrix through physical encapsulation, ion exchange, and precipitation mechanisms. The aluminosilicate structure of the geopolymer can create a stable environment in which these metals are tightly bound, preventing their leaching into the environment and thereby mitigating their toxic effects [37,52]. The immobilization of cations is often facilitated by the high surface area and ion exchange capacity of the geopolymer, which allows efficient bonding to metal ions through electrostatic attraction [4].

However, the immobilization of anionic species, such as chromium (Cr), is more challenging due to the different chemical behavior of these elements, which is influenced by their oxidation states. Chromium presents a further challenge due to its dual oxidation states: Cr³⁺ (chromium(III)) and Cr⁶⁺ (chromium(VI)). Cr³⁺ is less soluble and tends to form stable complexes with the geopolymer matrix, making it easier to immobilize. In contrast, Cr⁶⁺ is highly soluble, and in the form of chromate ions (CrO_4_²⁻), it is more mobile and less likely to be effectively immobilized by geopolymers. The inability of geopolymers to efficiently trap and stabilize Cr⁶⁺ is a major concern, as this form of chromium is more toxic and poses a significant environmental hazard [37,41].

The performance of geopolymers in immobilizing different metals is therefore not uniform and is highly dependent on the oxidation state and speciation of the metal in question. The changes in the oxidation state that occur under different environmental conditions, such as pH fluctuations, can alter the solubility and reactivity of metals, making them more difficult to immobilize. For example, in acidic environments, metals, such as chromium can reduce from Cr⁶⁺ to Cr³⁺, which is less mobile and easier to immobilize [9,52]. This variability in metal behavior complicates the design of geopolymers that can effectively immobilize a wide range of heavy metals under different environmental conditions.

In addition to the challenges posed by metal speciation, geopolymers also face limitations in terms of long-term stability, particularly under extreme environmental conditions. While geopolymers generally show promising results in controlled laboratory conditions, their effectiveness in real-world applications, such as wastewater treatment, is influenced by factors, such as the temperature, pH, and presence of other contaminants. Geopolymers can be susceptible to degradation under highly acidic or saline conditions, which can lead to the dissolution of the aluminosilicate network, releasing immobilized metals and reducing the effectiveness of the geopolymer [37,38]. 

In addition, while geopolymers perform well under controlled conditions, their scalability and real-world application on a large scale remain key challenges. Furthermore, the cost of producing geopolymers on a large scale and the need to optimize their performance for specific heavy metals and environmental conditions, adds to the complexity of their use. Further research is needed to address these challenges, including the development of geopolymer composites with enhanced stability, improved adsorption capacity, and greater adaptability to different environmental conditions [4,37].

### 2.3. Factors Affecting Encapsulation or Adsorption

The immobilization of heavy metals in geopolymers, whether via solidification/stabilization (S/S) or adsorption, is influenced by several key factors, including the geopolymer composition, surface area, porosity, curing conditions, and mechanical strength. The chemical composition of geopolymers, particularly the aluminosilicate matrix formed from materials, such as fly ash or metakaolin, plays a critical role in determining how well metals are immobilized. As mentioned above, cationic metals, such as lead (Pb²⁺) and zinc (Zn²⁺), tend to bind effectively to the geopolymer matrix, whereas anionic species, such as arsenate (As⁵⁺) and chromate (Cr⁶⁺), are more challenging to immobilize due to electrostatic repulsion [37,41].

- The surface area and porosity of the geopolymer significantly impact the immobilization process. Geopolymers with higher surface areas provide more adsorption sites for metal ions, which increases their ability to trap contaminants. For example, fly-ash-based geopolymers, with surface areas ranging from 56.0 to 100.99 m²/g, have shown superior adsorption capacity for heavy metals [4]. The porosity of the material also determines how well metals can be retained; geopolymers with mesoporous structures (pores ranging from 2 to 50 nm) tend to show better performance in adsorbing metals, as these pores are small enough to effectively trap metal ions [4,37]. However, an excessive increase in porosity can lead to a reduction in mechanical strength, which is critical for the long-term stability of the geopolymer matrix under environmental stress [33].
- Curing conditions, including the temperature and duration, are critical factors in determining the final properties of the geopolymer. Higher curing temperatures, typically in the range of 60 to 100 °C, promote the formation of a more compact and stable matrix, improving the geopolymer’s ability to immobilize metals [37,52]. Curing also reduces the porosity of the material, making it less likely to release trapped metals under extreme conditions. This process increases the overall stability and durability of the geopolymer in both solidification and adsorption applications. For example, geopolymers cured at temperatures above 80 °C typically exhibit improved mechanical strength, which is important for ensuring the long-term retention of metals [4,35].
- Mechanical strength is another essential factor in the immobilization of heavy metals. Geopolymers with higher mechanical strength are better able to withstand external stresses and prevent the release of trapped metals. The compressive strength of geopolymers can vary significantly depending on the raw materials used, curing conditions, and activator concentration. For example, metakaolin-based geopolymers have been shown to achieve compressive strengths between 30 and 80 MPa, while fly-ash-based geopolymers generally exhibit compressive strengths between 10 MPa and 40 MPa, depending on the synthesis conditions [4,35]. Higher curing temperatures tend to increase the mechanical strength of the geopolymer by promoting better polycondensation of the aluminosilicate framework.

In summary, it is essential to optimize the composition, surface area, porosity, curing conditions, and mechanical strength of geopolymers to improve their performance in immobilizing and adsorbing of heavy metals. 

### 2.4. Cases of Study Applications of Geopolymers in Heavy Metal Stabilization

The applications of geopolymers for heavy metal immobilization are diverse [14] and include four main areas:

- Treatment of fly ash from municipal solid waste incineration (MSWI): Fly ash from municipal solid waste incineration (MSWI-FA) contains hazardous heavy metals, such as Pb, Cd, Cr, Cu, Ni, and Zn, which can be leached into the environment and pose significant risks. The chemical composition of MSWI-FA often shows elevated concentrations of these metals. For example, the concentration of zinc (Zn) in MSWI-FA is often the highest, reaching up to 233.58 ppm in electrofilter ash (EF), while lead (Pb) and copper (Cu) can also reach significant levels, such as 60.80 ppm for Pb and 48.22 ppm for Cu [44,53,54]. The leaching of these metals can exceed the acceptable limits, making their stabilization critical for environmental protection.

Geopolymers have proven effective in immobilizing these heavy metals, particularly by incorporating MSWI-FA into an aluminosilicate matrix. This matrix either physically encapsulates or chemically binds the metals, reducing their mobility and leachability. In a study by Lancellotti et al. (2010), geopolymers incorporating 20% electrofilter fly ash (GPEF20) resulted in a non-detectable Pb leaching concentration, highlighting the effective immobilization of Pb [55]. Cd leaching was also negligible, with no detectable Cd released after leaching tests. Cr, however, showed a higher release, with concentrations up to 0.09 ppm in GPEF20, but still below hazardous waste limits [55].

In another case, a geopolymer synthesized with MSWI-FA and steel slag showed a significant reduction in the leaching of heavy metals, such as Pb and Cd. Specifically, the leaching concentration of Pb was reduced to 0.03 ppm in geopolymer matrices, and Cd remained undetectable, demonstrating the effectiveness of this approach for heavy metal immobilization [54].

Furthermore, a study by Liu et al. (2023) incorporating MSWI FA with steel slag showed that Zn leaching was significantly reduced when compared to untreated fly ash. The concentration of Zn in the leachate decreased from an initial value of 233.58 ppm in untreated MSWI-FA to 6.469 ppm in geopolymers containing 20% steel slag (N11SS20) [54]. The geopolymer also showed impressive immobilization efficiency for metals, such as Cr and Cu, with Cr leachate concentrations reduced to 1.00 ppm compared to 10.43 ppm in the untreated fly ash [54].

These results demonstrate that geopolymers can effectively immobilize heavy metals at concentrations well below the threshold for disposal in non-dangerous waste landfills, making them a promising and environmentally friendly solution for the stabilization of MSWI-FA. The alkaline environment during geopolymer synthesis promotes the precipitation of metals as hydroxides or encapsulates them within the gel network, further reducing their leachability [53,54].

- Wastewater treatment: Recent studies have explored the potential of geopolymers, derived from both coal fly ash and metakaolin, as effective adsorbents for the removal of heavy metals from wastewater. In various case studies, the use of fly ash-based geopolymers has demonstrated remarkable results in the removal of toxic metals from wastewater. For example, fly-ash-derived geopolymers were tested for the adsorption of lead and achieved a maximum removal efficiency of 174.34 mg/g, with optimum conditions set at pH 5, a contact time of 2 h, and an adsorbent dosage of 1.5 g/L [48]. Similarly, the removal efficiency of copper was improved using fly-ash-based geopolymers, with a reported maximum adsorption capacity of 152 mg/g at 45 °C [56]. These results highlight the effectiveness of fly-ash-based geopolymers in removing heavy metals from wastewater, which often contains high concentrations of toxic metals, such as Cu, Pb ,and Cd, with typical levels in industrial effluents ranging from a few ppb to several mg/L [48,49,56].

Metakaolin-based geopolymers have also shown strong potential for heavy metal adsorption, particularly for metals, such as copper and chromium. Metakaolin, when activated with sodium hydroxide or sodium silicate, forms a highly porous and stable structure that enhances its adsorptive properties. One study found that metakaolin-based geopolymers could effectively remove copper, with a maximum adsorption capacity of 152 mg/g [49,51]. In addition, metakaolin-based adsorbents have demonstrated significant capacity for the removal of chromium and lead, further emphasizing their versatility and potential in the treatment of a range of wastewater contaminants [48,51].

As reported in the previous paragraph, the chemical composition and structural characteristics of geopolymers, such as their high surface area and negative charge, play a crucial role in their ability to adsorb metal ions. Specific adsorption capacities vary depending on factors, such as the pH of the wastewater, temperature, and the type of metal to be removed. Typically, the adsorption follows isotherms, such as Langmuir and Freundlich, indicating both monolayer and multilayer adsorption mechanisms [48,56]. Furthermore, the use of sodium-based activators in metakaolin-based geopolymers has been shown to improve metal ion retention compared to potassium-based activators, making sodium-activated geopolymers a promising material for efficient metal removal [48,56].

- Landfill and soil remediation: In investigating the stabilization and solidification of landfill soils contaminated with heavy metals, several studies have highlighted the effectiveness of geopolymers, particularly those produced from fly ash (FA) and ground granulated blast furnace slag (GGBS), in immobilizing these contaminants. In one study, the concentration of heavy metals, such as arsenic (As) in untreated soil, was found to be 234 μg/L [57]. However, when treated with alkaline-activated slag, the concentration of leached arsenic was significantly reduced, demonstrating the ability of geopolymers to reduce leachability and effectively bind metals. The concentration of heavy metal in leachates after treatment was reduced by up to 92% after 28 days of curing, as was shown in the case of arsenic [57]. Similarly, in other studies focusing on different metals, such as lead and cadmium, the immobilization rate in treated soils reached up to 99.94% for Cd and 94.67% for Pb [58,59]. This efficiency in heavy metal reduction was directly correlated with the increase in the binder content (FA + GGBS) and curing time, which enhanced the ultimate compressive strength (UCS) of the solidified soils. The UCS of treated soils increased steadily with a increasing FA + GGBS content and curing time, reaching up to 19 MPa in some cases [59]. In addition, the presence of key chemical products, such as C-A-S-H and N-A-S-H gels, contributed to a denser soil structure, as observed in microscopic analyses using scanning electron microscopy (SEM) and X-ray diffraction (XRD) tests [57,59]. The pH-dependent leachability tests further confirmed that the leaching of metals was strongly influenced by the pH of the leachate, with a significant reduction in leachability at pH levels around 10, particularly for Cd [59].
- Nuclear material encapsulation: Geopolymers have emerged as an effective alternative to traditional cement for stabilizing hazardous radioactive waste. Cesium, a major radioactive contaminant, presents a particular challenge due to its high mobility. In geopolymer-stabilized matrices, Cs concentrations typically range from 0.5% to 2%, with leaching rates as low as 0.5% of the initial concentration, indicating an immobilization efficiency of up to 96% [13]. This is a significant improvement over conventional cement, where leaching rates can exceed 30% [34].

Strontium is also a challenge in radioactive waste disposal. In geopolymer matrices, Sr is primarily immobilized through the formation of insoluble precipitates, such as strontium hydroxide and carbonate. The concentration of Sr in waste is typically between 1 and 3%, and leaching studies have shown that geopolymers can reduce Sr leaching to below 3%, achieving an immobilization efficiency greater than 97% [34]. This performance exceeds that of conventional cement, where leaching can exceed 10% [13,60].

Furthermore, the incorporation of additives, such as zeolites, or modifications to the Si/Al ratio further enhance immobilization, particularly for Cs and Sr. Geopolymer ceramics, formed under high-temperature conditions, offer even greater stabilization by encapsulating radionuclides, such as cesium and strontium, in crystalline phases, such as tobermorite and xonotlite, further improving leach resistance [13,34].

## 3. Introduction to Stabilizing Agents in Heavy Metal Immobilization

Stabilizing agents play a crucial role in immobilizing heavy metals within waste materials to prevent their leaching and release into the environment. The use of specific agents, such as organic and inorganic chelating agents, phosphates, sulfides, and dithiocarbamates, effectively reduces the mobility of metals, such as Pb, Cd, Zn, and Cr, within matrices, such as municipal solid waste incineration fly ash (MSWI-FA) and contaminated soils [61].

Inorganic stabilizers, including phosphates and sulfides, convert heavy metals into less soluble forms. For example, sodium sulfide (Na_2_S) effectively converts metals into insoluble forms, such as metal sulfides, thereby reducing leachability [52,61]. Similarly, phosphates, such as sodium dihydrogen phosphate (NaH_2_PO_4_), form stable metal-phosphate compounds that resist dissolution, minimizing the release of hazardous metals into leachates [61].

### 3.1. Mechanisms of Stabilization

The stabilization mechanisms vary depending on the type of agent and metal involved. Organic and inorganic agents can employ different strategies, including chemical precipitation, ion exchange, reduction, and complexation.

#### 3.1.1. Solidification/Stabilization (S/S) with Stabilizing Agents

Solidification/stabilization (S/S) immobilizes heavy metals by incorporating them into a stable matrix, often cementitious materials. The hydration process within cementitious matrices, for example, forms compounds, such as calcium silicate hydrate, that encapsulate metals and reduce their mobility [52]. Cementitious stabilization is commonly used but is sometimes limited by volume expansion and the secondary release of contaminants when the matrix is exposed to acidic environments.

#### 3.1.2. Adsorption and Chelation

**Figure 3:**
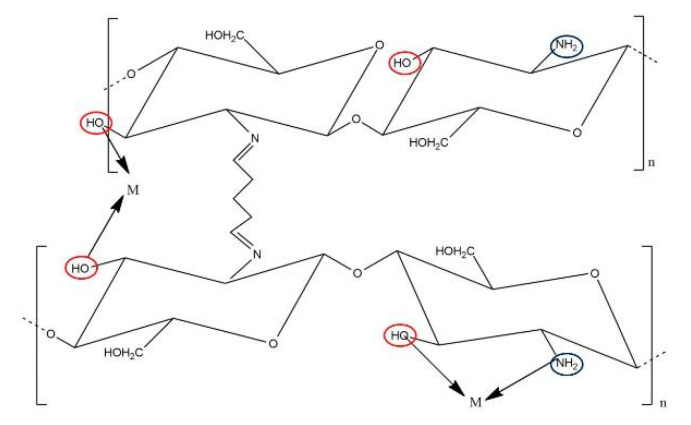
Chitosan chelation mechanism for metal ions.

Organic chelators, such as dithiocarbamate (DTC) compounds, use sulfur-based groups to form stable complexes with heavy metals. DTCs are particularly effective in the treatment of MSWI-FA, where they form macromolecular chelates that embed heavy metals in a three-dimensional network structure, thereby enhancing long-term stabilization [62]. Chitosan and modified chitosan are also used for adsorption (Figure 3), providing an environmentally friendly option due to chitosan’s biocompatibility and high metal binding efficiency through amino and hydroxyl functional groups [63].

Chelation stabilizers often target specific functional groups in metals, resulting in the formation of stable compounds that resist leaching under varying environmental pH conditions. For example, thiourea and dithiocarbamates (DTCs) are sulfur-containing agents that immobilize metals, such as Cd, Pb, Cr, and Ni, in forms resistant to acidic leachates. This property is particularly valuable in landfill leachate management, where a ternary chelating system combining TMT (trithiocyanuric acid trisodium salt) and NaH_2_PO_4_ demonstrated stabilization rates for Cd and Pb exceeding 99%. Such ternary systems effectively reduce the leaching toxicity of heavy metals, outperforming single agents in complex matrices like municipal solid waste incineration fly ash (MSWI-FA) [61].

Moreover, piperazine-based chelating agents, including derivatives of DTC and other macromolecular stabilizers, show exceptional immobilization efficiency [64]. These agents form robust metal-chelate bonds that remain stable across a wide pH range, particularly for amphoteric metals, such as Pb and Zn. Notably, under simulated acidic rain conditions, TS-300, a piperazine-based chelator, reduced Pb leaching by up to 98%, even at low dosages (1.5%). This was attributed to the formation of chain-like macromolecular complexes that limit the mobility of metals. However, environmental risk assessments highlight that prolonged storage or extreme pH conditions can lead to the partial re-release of stabilized metals, emphasizing the need for careful long-term management strategies.

The synergistic use of inorganic (e.g., Na_2_S, phosphates) and organic chelators has emerged as a promising method to enhance stabilization performance. Inorganic agents, such as Na_2_S, convert metals into sulfide precipitates, which are less soluble, while organic chelators provide additional coordination sites for stabilization [61]. Combined systems, such as TMT–Na_2_S–thiourea, are particularly effective for circulating fluidized bed fly ash (CFB-FA), achieving the near-complete immobilization of Cd and Pb.

These findings underline the importance of selecting tailored chelation systems that account for both the metal species and environmental conditions to ensure minimal leaching risks and long-term stabilization in landfill applications. Future research should focus on optimizing chelator combinations and evaluating their stability over extended periods under variable environmental stressors.

### 3.2. Applications of Stabilizing Agents in Heavy Metal Treatment

Stabilizing agents are used in a variety of heavy metal remediation situations, particularly in the treatment of incinerator fly ash, contaminated soils, and industrial by-products. In landfill applications, both cementitious and organic chelating agents are commonly used to ensure the immobilization of heavy metals, such as Pb and Cd, which are prone to leaching. By forming highly stable bonds, these agents enable the treated waste to meet stringent landfill standards, ensuring long-term environmental safety [65]. In addition, agents, such as sodium sulfide, are applied to industrial wastes for their ability to convert soluble metal species into non-leachable forms, reducing the potential for groundwater contamination [61,66].

In summary, stabilizing agents are essential in environmental management for the treatment of hazardous waste containing heavy metals. Their selection and application depend on the specific environmental requirements and waste characteristics, ensuring that heavy metals are effectively immobilized, thus preventing environmental and health risks associated with metal leaching.

## 4. Utilization of Stabilizing Agents in Geopolymers for Heavy Metal Immobilization

The aim of this review is to analyze various applications of chemical stabilizers, both organic and inorganic, with different stabilization mechanisms (such as reduction, chelation, and ion exchange), within geopolymers. The focus is on evaluating whether the addition of these chemical agents improves the stabilization/solidification and adsorption of heavy metals derived from industrial waste. This is critical as the presence of such contaminants, particularly in municipal solid waste incineration fly ash, poses significant environmental and health risks. 

The incorporation of stabilizing agents into geopolymers has become a key development in improving the immobilization of heavy metals. This approach addresses the need for improved leaching resistance and binding efficiency for metals, such as Pb, Cd, Cr and Cu. Stabilizing agents interact with the aluminosilicate structure of geopolymers, either chemically or physically, to create immobilizing environments for heavy metals through solidification/stabilization (S/S) and adsorption mechanisms. Recent research has investigated a wide range of stabilizing agents, organic chelating agents, such as dithiocarbamate (DTC) ,and inorganic compounds, revealing different mechanisms and impressive immobilization efficiencies under different environmental conditions [67]. 

**Figure 4:**
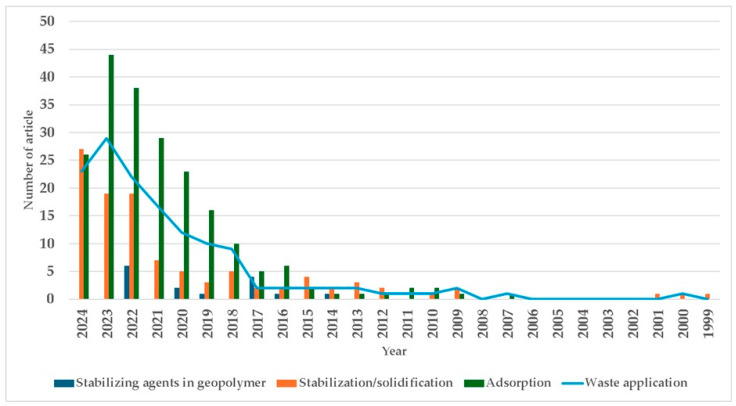
Trends in scientific publications on stabilizing agents in geopolymer, encapsulation (S/S), adsorption, and waste applications (1999–2024).

Figure 4 shows the increase in published papers focusing on the use of stabilizers in geopolymers to promote metal immobilization. A significant increase can be observed since 2018.

### 4.1. Solidification/Stabilization (S/S)

In the studies reviewed, various chemical agents were used to enhance the stabilization and solidification of a broad range of heavy metals, including chromium (Cr), copper (Cu), lead (Pb), cadmium (Cd), and nickel (Ni) in geopolymers. These heavy metals are often found in hazardous waste materials, such as municipal solid waste incineration fly ash (MSWI-FA), and pose significant environmental risks due to their high toxicity and mobility. Among the agents studied, reducing agents, chelating agents, and precipitating agents were identified as highly effective in reducing the leachability and enhancing the immobilization of these metals.

The use of sodium sulfide (Na_2_S) as a reducing agent was particularly notable in the reduction of Cr(VI) to Cr(III), a much less toxic and less soluble form of chromium [45,67,68,69]. Na_2_S serves as a reductant, converting Cr(VI) into the more stable Cr(III), which is then chemically incorporated into the geopolymer matrix through electrostatic attraction to negatively charged [AlO_4_]^−^ units. This reduction process not only decreased the leachability of Cr but also contributed to the stabilization of other heavy metals, such as Cu, Pb, Ni, and Cd [45]. The addition of Na_2_S to fly-ash-based or metakaolin-based geopolymers resulted in a remarkable reduction in chromium leaching, with concentrations consistently reduced to levels below the regulatory limits [68,69]. The study by Sun et al. [68] investigated the detoxification and immobilization of Chromite Ore Processing Residue (COPR), a waste produced in the high lime chromium salt production process using chromite containing a fraction of leachable Cr(VI), with a metakaolin-based-geopolymer-added sodium sulfide. Without the addition of S^2−^, the leached Cr (VI) and Cr_tot_ are more than 45 ppm, with S^2−^ being added with a molar ratio S^2−^/Cr (VI) = 10, leached Cr (VI) and Cr_tot_ are 0.01 ppm and 1.37 ppm, respectively, reducing the leaching concentrations by 99% and 97%. The mechanism analysis shows that Cr^3+^ was attached and immobilized by the aluminum-oxygen unit ([–OAl(OH)_3_]^−^) of the geopolymer [68]. In another study, Zhang et al. investigated the addition of S^2−^ to fly ash geopolymer formulations to be used in the immobilization of Cr(VI) added as Na_2_CrO_4_ e PbCrO_4_, with the aim of reducing much of the Cr(VI) to Cr(III) and hence improving the observed immobilization performance [69]. It was observed that in leaching in deionized water and mineral salts (Na_2_CO_3_ and MgSO_4_), stabilization increased from less than 20% without the stabilizer to more than 99.9% with the stabilizer. The improvement in leaching performance in a sulfuric acid medium is nowhere near as great as is observed in any of the other cases, reducing the immobilization efficiency to around 80%, which is still much better than the 10% immobilization observed in H_2_SO_4_ without Na_2_S, most probably due to the oxidation of the Cr(III) to Cr(VI) [68,69].

3S^2−^ + 8CrO_4_^2−^ + 20H_2_O → 3SO_4_^2−^ + 8Cr^3+^ + 40OH^−^

3S^2−^ + 4Cr_2_O_7_^2−^ + 16H_2_O → 3SO_4_^2−^ + 8Cr^3+^ + 32OH^−^

This reduction in leachability was not limited to chromium alone; the leachability of Cu, Cd, Pb, and Ni was similarly reduced, showing the broad applicability of Na_2_S in stabilizing multiple heavy metals [45]. These metals are likely to be retained to a greater extent within the geopolymeric matrix, as the addition of sodium sulfide is likely to form insoluble precipitates, such as sulfides.

The study conducted by Xu et al. explored the combined approach of geopolymerization and chemical stabilizers for treating heavy metals in MSWI-FA. The leaching tests revealed that the concentrations of Cu, Cr, Cd, Ni, and Pb met the disposal standard requirements, with over 99.8% of these metals being effectively stabilized. Notably, Cr and Ni were found to be below the detection limits, highlighting the crucial role of Na2S in converting Cr(VI) to Cr(III), which significantly reduces its mobility [45].Similarly, ferrous-based reductants facilitate the reduction of Cr(VI) to Cr(III) and enable its structural stabilization within metakaolin-based geopolymer matrices [70,71]. In particular, the addition of FeCl_2_ was studied by Chen et al. and was found to be highly effective in reducing Cr(VI) to Cr(III), resulting in a significant reduction in leachability [71]. By adding potassium dichromate (K_2_Cr_2_O_7_) and ferrous chloride to the base colloid formed by mixing metakaolin and the alkaline activator, a Cr(VI)-contaminated metakaolin-based geopolymer was prepared. When different ratios between Cr(VI) and Fe^2+^ were evaluated, the FeCl_2_ addition increased from 0.5 to1.5 wt% with the constant addition (0.1 wt%) of Cr (VI), and the leached Cr_tot_ samples decreased, from 20.05 to 0.42 ppm. When the addition of FeCl_2_·4H_2_O was further increased from 1.5 to 3.0 wt%, leached Cr_tot_ fluctuated slightly, from 0.42 to 0.135 ppm. The maximum chromium capacity in the geopolymer was found to be 0.8 wt%, achieved with an optimal Fe^2+^/Cr(VI) molar ratio of 4:1. This ratio ensured that the leached chromium concentration remained below 5 mg/L [71]. Based on XRD, SEM, and XPS analyses, the mechanism behind the detoxification and immobilization of Cr was proposed and explained. Initially, negatively charged [AlO4]- groups were formed through geopolymerization, while Cr(VI) was reduced to Cr^3+^ with the help of Fe^2+^. Subsequently, Cr^3+^ was attracted to [AlO4]^−^ due to electrostatic forces and became stabilized within the geopolymer structure. Hence, the immobilization of Cr(VI) in geopolymers involved both a redox reaction and a fixation process [71].

3Fe^2+^ + Cr^6+^ → 3Fe^3+^ + Cr^3+^

In a separate study, Chen et al. investigated the reduction and immobilization of Cr(VI) in a metakaolin-based geopolymer using FeSO_4_·7H_2_O as the reducing agent, applying two different methods: A) Cr(VI) was reduced and immobilized simultaneously within the geopolymerization system containing FeSO^4^·7H_2_O; B) Cr(VI) was first reduced in the FeSO_4_·7H_2_O solution and subsequently immobilized in the geopolymerization system. The molar ratio of Fe(II)/Cr(VI) was set to 3.5 in both processes [70]. In process A, the chromium immobilization in the geopolymer was limited to 0.3 wt.%, while in process B, the immobilization reached up to 0.7 wt.%. These differences were attributed to the redox reaction mechanisms. Process A achieved synchronous reduction and immobilization of Cr(VI) in the geopolymerization system, whereas process B resulted in better immobilization efficiency and a higher immobilization amount [70]. For the leachate with 0.1 wt.% Cr (VI) and without Fe (II) addition, Cr_tot_ and Cr (VI) were 46.9133 ppm and 39.890 ppm. With Fe (II) addition, Cr_tot_ and Cr (VI) leaching values decreased 0.4576 ppm and 0.057 ppm, respectively. Leached Fe was 0.0469 ppm [70].

**Figure 5:**
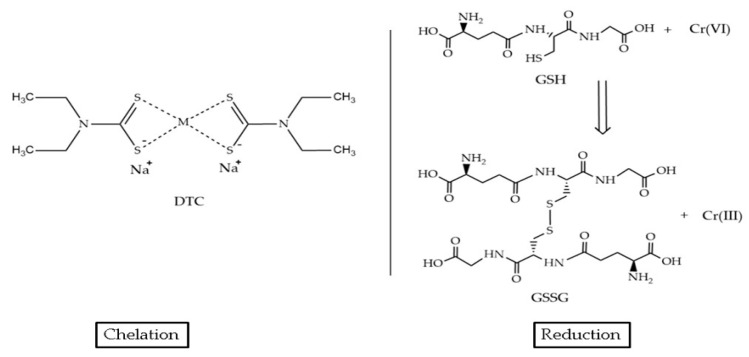
Chelation via dithiocarbamate DTC and reduction of chromium (VI) using glutathione GSH mechanisms.

Furthermore, for chromium-containing sludge (CCS), a solid hazardous waste containing various heavy metals, CCS was solidified/stabilized through a combined method of the glutathione (GSH) pre-reduction of Cr(VI) and alkali-excited blast furnace slag (BFS) preparation of the geopolymer [72]. GSH pre-reduction technology significantly increases the maximum dosage of CCS in the solidified body and increases the proportion of heavy metals in the residual state. The reduction mechanism is presented in Figure 5. The maximum content that can meet the Toxicity Characteristic Leaching Procedure TCLP standard is 2.5% when CCS is solidified without pre-reduction experiments, and the maximum content is 5% after pre-reduction. Overall, the addition of GSH doubled the maximum dosage of CCS. The GSH pre-reduction process significantly enhances the conversion of Cr(VI) to Cr(III), which is subsequently stabilized in the geopolymer matrix through chemical encapsulation and physical entrapment (Figure 5) [72]. The solidified products meet landfill disposal standards and exhibit promising mechanical properties for use in construction materials [72].

In addition to reducing agents, chelating agents, such as TBP (C_12_H_27_O_4_P, tributyl phosphate),DTC (C_5_H_10_NS_2_Na, Sodium diethyl dithiocarbamate), TMT (C_3_N_3_Na_3_S_3_ (2, 4, 6-trithione-1, 3, 5-triazine trisodium salt), and SGA (C_4_H_5_N_3_S_6_, sixthio guanidine acid), were explored for their role in stabilizing heavy metals within the geopolymer matrix [45,67]. Chelating agents function by forming stable complexes with metal ions, effectively reducing their solubility and mobility.

**Figure 6:**
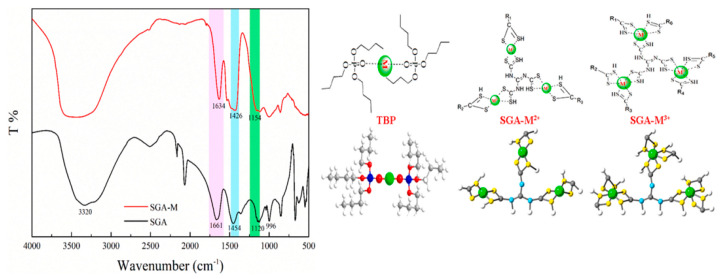
(**left**) FT-IR of SGA before and after curing. (**right**) The structures of TBP (tributyl phosphate) and SGA (sixthio guanidine acid) chelated with heavy metals reprinted with permission from [45]. Copyright 2022, Elsevier.

The use of TBP and SGA in the treatment of municipal solid waste incineration fly ash, with a combination of geopolymers and organic/inorganic chemical agents, has been used to immobilize heavy metals, including copper (Cu), cadmium (Cd), nickel (Ni), chromium (Cr), and lead (Pb) [45]. In this study, the MSWI-FA underwent activation through the geopolymerization process, resulting in the formation of Al/Si-based hydration products. Simultaneously, the chemical stabilizers interacted with the heavy metals to form chelates, providing a dual effect in stabilizing the metals. The oxygen atom in TBP and the sulfur atom in SGA coordination groups possess a large atomic radius and low electronegativity, making them highly polarizable [45]. This characteristic allows them to generate a negative electric field and effectively donate electrons. Heavy metal ions, which typically have vacant d orbitals, are readily able to accept electrons or coordination groups, forming coordination bonds. Consequently, both the oxygen in TBP and sulfur in SGA can easily coordinate with heavy metal ions [45]. When SGA interacts with heavy metals, changes can be observed in its infrared spectrum. For example, the peak at 3320 cm^−1^ corresponds to the stretching vibration of the -N-H group, while the absorption at 1661 cm^−1^ is attributed to the stretching vibration of the -C-N group. Additionally, the peaks at 1454 cm^−1^ and 1120 cm^−1^ represent the stretching vibrations of -N-C-S and -C-S, respectively. The shift of these peaks, especially at 1454, 1120, and 996 cm^−1^, indicates that SGA undergoes a chelation reaction with heavy metals (Figure 6a,b) [45]. The MSWI-FA was processed using a combination of geopolymerization and chemical stabilizers, and the concentrations of all six heavy metals remained within the corresponding regulatory standards at 7 days, 14 days, and 30 days. Throughout the treatment, only small amounts of chemical reagents were added, which effectively contributed to the stabilization of the heavy metals. This approach demonstrated the efficacy of using minimal chemical additives in ensuring the long-term immobilization of hazardous metals in MSWI-FA, maintaining environmental safety standards over time. This significantly improved the immobilization of Pb, Cu, Ni, and Cd [45]. 

In the case of fly-ash-based geopolymers, DTC (dithiocarbamate) and TMT (2, 4, 6-trithione-1, 3, 5-triazine trisodium salt) have been used as detoxifying agents [68]. In this study, the use of fly ash as a precursor for geopolymers has been explored, and the effects of different dosages and chemical valences of chromium reagents on the geopolymer’s performance, as well as the detoxification efficiency of the chemical agents, have been examined. A chromium reagent Cr(NO_3_)_3_ was incorporated into the fly-ash-based geopolymers at dosages of 0%, 0.5%, 1.0%, 1.5%, and 3.0%. It was found that when the dosage of Cr(NO_3_)_3_ reached 3.0%, the leaching toxicity of the resulting geopolymer F-Cr(NO_3_)_3_-3.0 (where F represents a geopolymer based on Fly Ash) was 1.93 mg/L, which is 27.6 times higher than F-Cr(NO_3_)_3_-1.0. Despite this increase, the value remained within the threshold limit for the total chromium content [67].

After adding the four chemical agents, the performance of the geopolymers showed different levels of improvement. TMT exhibited the best performance compared to DTC, both of which are organic chemical agents. Leached Cr_tot_ without stabilizers containing 3% Cr (NO_3_)_3_ is 1.93 mg/L, and by adding TMT and DTC, leaching values were, respectively, 0.59 and 1.05 mg/L. In the same study, it was added in the same proportion, and Na_2_S and NaH_2_PO_4_ (as precipitating agent) leaching values were, respectively, 1.27 and 1.70 mg/L. The addition of a reducing agent, such as Na_2_S is highly effective in reducing the leaching of Cr when it is present as Cr(VI) [45,68,69]. Organic agents are more effective than inorganic ones in the geopolymer stabilization process. Cr(III) is highly prone to oxidation back to Cr(VI), which increases its environmental toxicity. The sulfur atom in the thiol group of TMT has an empty d orbital, which facilitates its ability to lose electrons and capture heavy metals through the negative electric field generated via polarization. This mechanism enables the reduction of Cr(VI) to Cr(III), which is then stabilized in the geopolymer matrix through complexation reactions [67]. DTC, with its similar sulfur-based functional groups (Figure 5), also showed positive results in leaching tests, indicating that sulfur-containing organic agents are particularly effective in reducing chromium toxicity. Therefore, organic chelating agents containing sulfur atoms, which can easily donate electrons, perform a dual function as both reducing and chelating agents. This is why organic chelates are more effective than inorganic chemical stabilizers [67].

The combination of geopolymers with chemical agents not only reduced the leachability of these metals but also enhanced the overall strength and durability of the treated materials. In several studies, the compressive strength of geopolymers remained high, often exceeding 30 MPa, even with the incorporation of heavy metals into the matrix [45,67,71]. This is an important consideration, as it indicates that the stabilization of heavy metals through the geopolymerization process does not compromise the structural integrity of the material. For example, in tests where Na_2_S and FeCl_2_ were added to geopolymers, the compressive strength was maintained at high levels while also achieving the significant stabilization of Cr, Cu, Pb, and Ni [45,71]. This dual benefit—enhancing both the mechanical properties and environmental safety of the material—makes this treatment method not only environmentally sound but also economically viable for potential use in construction applications [45,71].

In conclusion, the studies reviewed demonstrate the significant role of chemical agents in improving the solidification and stabilization of heavy metals, such as chromium, copper, lead, cadmium, and nickel in geopolymers. The combination of reducing agents, such as sodium sulfide (Na_2_S), and ferrous-based agents (FeCl_2_, FeSO_4_) has proven to be highly effective in reducing Cr(VI) to the less toxic and less soluble Cr(III), significantly reducing leachability and improving the immobilization of heavy metals [45,68,69,70,71]. Organic stabilizers, TMT, and DTC, have further enhanced the performance of geopolymers by forming stable complexes with metal ions, reducing their solubility and mobility [67]. The studies consistently show that organic chelating agents containing sulfur atoms, such as TMT, DTC, and SGA, outperform inorganic stabilizers by providing both reducing and chelating functions, thus improving the overall stabilization of heavy metals [45,67].

The combination of improved metal immobilization and retained mechanical strength makes this approach particularly beneficial for practical applications in environmental remediation and construction materials [45,67,71]. These findings emphasize the effectiveness of using geopolymers with chemical stabilizers for hazardous waste treatment and suggest their potential for large-scale applications in waste management and sustainable construction as shown in Table 1.

### 4.2. Adsorption

The adsorption-based stabilization of heavy metals in geopolymers uses organic and inorganic stabilizers to introduce or enhance active binding sites within the matrix [73,74,75]. These sites enable the physical and chemical capture of metal ions, primarily through surface complexation, ion exchange, and complex formation [76,77,78]. Recent studies have investigated the effectiveness of organic stabilizers, including biopolymers and surfactant-modified geopolymers, in capturing and immobilizing metals, such as Pb, Cd, Cu, and Pb from aqueous solutions [73,78].

In the study of Yan et al., novel composites of porous geopolymer, sodium alginate (C_6_H_7_NaO_6_)n, and chitosan (C_8_H_13_NO_5_)n and hydrogen peroxide (H_2_O_2_) were developed by combining a metakaolin-based geopolymer with a sodium alginate solution and chitosan, designed specifically for the direct removal of lead (Pb) from wastewater [73]. The findings indicate that the equilibrium adsorption capacity of the composites for Pb removal ranged from 120.45 to 142.67 mg/g at pH 5 and 25 °C, reaching 95% removal. The adsorption process followed the pseudo-second-order kinetic model and the Freundlich isotherm model. After adsorption, the composites retained the crystal structure of hydrocerussite (Pb_3_(CO_3_)_2_(OH)_2_) [73]. SEM images of the geopolymer composite after adsorption reveal the formation of new crystal growth on the surface during contact with Pb²⁺. After a certain period, a transformation into hydrocerussite occurs. This phenomenon can be explained by the functional groups of sodium alginate and chitosan dispersed in the geopolymer composite, which capture metal cations through adsorption via electrophilic–nucleophilic interactions and ion exchange [73]. Additionally, excess OH⁻ from the geopolymer leaches out to the surface through the pore network, promoting an irreversible crystallization process. Moreover, the porous structure of the geopolymer composite allows the adsorption process to occur both on the surface and internally, as ions penetrate the inner parts of the matrix. Pb²⁺ ions were strongly attracted by the functional groups from alginate–chitosan and were immobilized through crystallization between hydroxyl groups (OH⁻) from the geopolymer and dissolved CO_2_ from the air [73].

Sodium alginate was also utilized in the work conducted by Ge et al., where a novel hybrid sphere composed of a geopolymer and alginate (GAS) was created using a sustainable one-pot method, combining a metakaolin-based geopolymer and sodium alginate, and the hybrid spheres were formed after solidification and were kept in CaCl_2_ [78]. The GAS-4 (in which the mass ratio of the geopolymer and sodium alginate was 4) was used as an adsorbent for the removal of Cu(II) in water. GAS-4 demonstrated an excellent Cu(II) removal efficiency of 99 ± 3.4% from a 50 mg/L solution at a dosage of 0.15 g/100 mL. The adsorption kinetics were well described by the pseudo-second-order model, suggesting a chemical interaction between GAS-4 and Cu(II) [78]. The adsorption isotherm followed the Langmuir model, indicating monolayer adsorption of Cu(II) on GAS-4. The effect of the solution pH was studied, and the removal of Cu(II) by GAS-4 was highly pH-dependent, with higher pH values enhancing the Cu(II) removal efficiency. As the pH increased, the carboxyl groups were gradually deprotonated, leading to a significant enhancement in the Cu(II) adsorption efficiency, so an ion-exchange mechanism occurred throughout the adsorption process, involving the exchange between -COO⁻Na(I)/Ca(II) in GAS-4 and Cu(II) in water [78].

**Figure 7:**
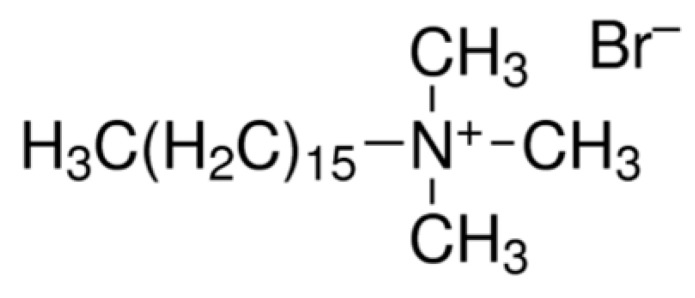
Chemical structure of the cationic surfactant cetyltrimethylammonium bromide (CTAB).

The cationic surfactant cetyltrimethylammonium bromide (CTAB) (Figure 7) has also been used to improve the adsorption capacity of geopolymers [74,77]. CTAB-modified geopolymers have a positively charged surface that attracts negatively charged metal complexes, significantly increasing Cr(VI) adsorption rates [74].

**Figure 8:**
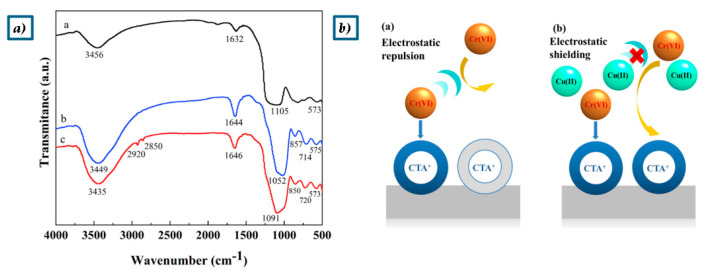
(**a**) The Fourier transform infrared (FTIR) spectra of metakaolin (MK) (a), geopolymer (GP) (b), and geopolymer-cetyltrimethylammonium bromide (GP-CTAB) (c). (**b**) Shielding effect of Cu(II) on the electrostatic repulsion between Cr(VI): (a) without Cu(II) and (b) with Cu(II). When the color of the CTA+ group changes from grey to blue, the adsorption site becomes available adapted from [75].

In the work of Yu et al., a metakaolin-based mesoporous geopolymer (GP-CTAB) was utilized as an adsorbent for Cu(II) and Cr(VI) with CTAB serving as an organic modifier [74]. The use of GP-CTAB is studied for both the removal of metal anions and cations from aqueous solutions. GP-CTAB is capable of simultaneously adsorbing anions without compromising its ability to adsorb heavy metal cations, offering an advantage over traditional geopolymers. When the FT-IR spectra of GP and GP-CTAB are compared (Figure 8a), the absorption peaks at 2920 cm⁻¹ and 2850 cm⁻¹ in GP-CTAB indicate the presence of -CHn, confirming the presence of quaternary ammonium salt cations (CTA^+^) [74]. Meanwhile, the characteristic peaks of the GP remain intact in GP-CTAB, and overall, the absorption peaks show minimal changes. This demonstrates that despite the organic modification, the addition of CTA^+^ does not alter the fundamental nature of the geopolymer, and GP-CTAB still belongs to the geopolymer category [74]. After organic modification, the resulting product remains classified as a geopolymer, distinguishing it from traditional geopolymers by the presence of a quaternary ammonium salt cation attached to its surface. The maximum theoretical adsorption capacities of GP-CTAB for Cu(II) and Cr(VI) in the binary system were found to be 108.2 mg/g (92%) and 95.3 mg/g (90%), respectively [74]. It was also observed that the presence of Cu(II) in the solution enhanced the adsorption of Cr(VI). In the Cu(II)/Cr(VI) binary system, the adsorption of Cu(II) is slightly hindered by the presence of interfering ions (Na^+^ and K^+^), while the adsorption of Cr(VI) may be enhanced due to electrostatic shielding effects caused by unadsorbed Cu(II) in the solution (Figure 8b) [74]. 

In another work, Singhal et al. synthesized a nanoporous metakaolin-based geopolymer with the use of CTAB [77]. The experimental results verified that the geopolymer could adsorb copper ions completely at lower concentrations and partially at higher concentrations. The addition of CTAB significantly alters the porosity and surface area. The surface area increases from 137 to 216 m²/g, while the pore volume increases from 0.19 to 0.22 cm³/g [77]. It was observed that up to 120 ppm of copper ions can be fully adsorbed onto the nanoporous geopolymer. However, higher concentrations could not be completely removed, as the nanoporous geopolymer reached its saturation adsorption capacity. A high adsorption capacity of 1.65 m/g was achieved with the nanoporous geopolymer, which is significantly higher and/or comparable to those reported in the literature [77]. 

Another stabilizer, dithiocarbamate (DTC), a chelating agent, enhances adsorption by forming metal–sulfur complexes that strongly bind heavy metals, such as Cd [77]. 

**Figure 9:**
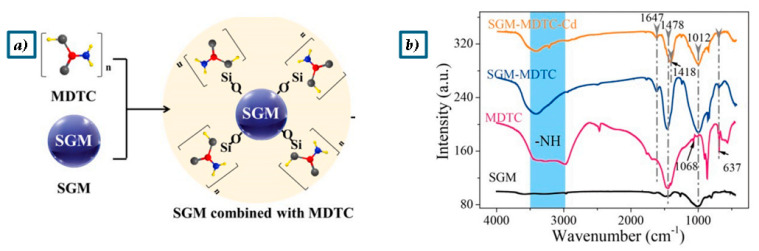
(**a**) SGM-MDTC (slag-based geopolymer microspheres grafted with macromolecular dithiocarbamate). (**b**) The FT-IR of SGM, MDTC, SGM-MDTC, and SGM-MDTC-Cd. Reprinted with permission from [77]. Copyright 2022, Elsevier.

Su et al. synthesized a novel composite adsorbent (SGM-MDTC) by grafting macromolecular dithiocarbamate (MDTC) onto slag-based geopolymer microspheres (SGMs), with the activation of Si–O–Si in SGM to enhance the Cd(II) adsorption performance of the geopolymer adsorbent (Figure 9a). The morphology of SGM and SGM-MDTC changed significantly, and the specific surface area of SGM increased from 52.99 to 72.36 m²/g after the process of MDTC anchoring. The adsorption capacity of the synthesized SGM-MDTC material (205.8 mg/g) was nearly twice as high as that of SGM (106.7 mg/g) under static adsorption conditions. Dynamic adsorption proved to be more suitable for practical applications than static adsorption, with the adsorption capacity of SGM-MDTC reaching as high as 382.8 mg/g. This demonstrated that the synthesized SGM-MDTC material exhibited excellent Cd(II) purification performance and high application potential. This sample was analyzed using FT-IR to confirm that SGM-MDTC could effectively adsorb Cd [76]. The FT-IR spectra of SGM-MDTC and SGM-MDTC-Cd showed peaks corresponding to the -OH stretching vibration at 3576 cm⁻¹ and bending vibration at 1647 cm⁻¹, attributed to the abundant hydroxyl groups in the geopolymer. These -OH peaks were not observed in the SGM profile as they were masked by other functional group peaks. The peak at 1012 cm⁻¹, found in all profiles, corresponded to the asymmetric stretching vibration of the T-O-Si unit, forming the geopolymer skeleton. Additionally, FT-IR spectra of MDTC showed characteristic peaks at 3276 and 2981 cm⁻¹ for -NH groups and peaks at 1465, 1068, and 637 cm⁻¹ corresponding to N-CS_2_, C–S, and C-S bonds. In the SGM-MDTC profile, the characteristic peaks at 1465 and 637 cm⁻¹ appeared, but the dithioamino group peaks were absent, likely due to overlap with strong peaks from the geopolymer. After Cd(II) adsorption, the 1465 cm⁻¹ peak shifted to 1418 cm⁻¹, indicating the formation of strong bonds between Cd(II) and SGM-MDTC [76] (Figure 9b). 

In conclusion, the studies highlight the significant advancements in the use of modified geopolymers and stabilizing agents for the adsorption and removal of heavy metals from aqueous solutions. The incorporation of organic and inorganic stabilizers, such as sodium alginate, chitosan, CTAB, and MDTC, enhanced the adsorption capacities of geopolymers for metals like Pb, Cu, Cr, and Cd [73,74,76,77,78]. These modifications not only increased the surface area and porosity of the geopolymers, improving metal ion uptake, but also promoted the formation of stable complexes and chemical interactions between the adsorbent and metal ions [73,77]. Moreover, the synergy between the stabilizers and geopolymers enabled the efficient simultaneous removal of metal cations and anions, with high removal efficiencies observed in both static and dynamic adsorption conditions [74,77]. The successful grafting of MDTC onto slag-based geopolymer microspheres demonstrated an excellent Cd(II) removal performance, reinforcing the high potential of these composite materials for practical applications in environmental remediation [76]. These findings demonstrate the effectiveness of geopolymer-based composites in enhancing the immobilization and stabilization of heavy metals, offering a promising solution for wastewater treatment and other environmental applications.

Table 2 presents a summary of the results discussed in the previous paragraph.

## 5. Conclusions

In conclusion, this review highlights the significant advances in the incorporation of chemical stabilizers into geopolymers, both in solidification/stabilization (S/S) and adsorption processes, for the effective immobilization and removal of heavy metals from solids and aqueous solutions. The incorporation of inorganic stabilizing agents, such as sodium sulfide (Na_2_S) [45,67,68,69], ferrous-based reductants (FeCl_2_ and FeSO_4_) [70,71], and organic chelating agents, like DTC and TMT [67], has been particularly successful in reducing the leachability of heavy metals, such as Cr, Cu, Pb, Cd, and Ni. These agents promote chemical reactions that convert toxic metal ions, such as Cr(VI), into less mobile and less toxic forms, thereby significantly improving the effectiveness of S/S. In addition, the synergistic effect of these chemical stabilizers and geopolymers has led to the improved encapsulation and immobilization of heavy metals through mechanisms, such as ion exchange, precipitation, and complex formation.

For adsorption, the modification of geopolymers with stabilizing agents, such as sodium alginate [73,78], chitosan [73], CTAB [74,77], and MDTC [76], has been shown to be highly effective in enhancing metal ion uptake. These modifications increase the surface area and porosity of the geopolymer, providing more adsorption sites and facilitating the formation of stable metal–ligand complexes. The ability of these composites to remove both metal cations and anions, with high efficiency observed over a range of pH and environmental conditions, demonstrates their versatility and potential for wastewater treatment and environmental remediation.

The combination of S/S and adsorption mechanisms within geopolymers, aided by chemical stabilizers, provides a robust approach to the long-term immobilization of hazardous metals, ensuring that they are contained and prevented from leaching into the environment. The success of these methods both in the laboratory and in potential real-world applications underlines their environmental benefits, offering a sustainable solution for the treatment of hazardous waste. The addition of stabilizing chemical agents to geopolymer formulations is therefore a particularly effective method of inerting difficult-to-treat-wastes using unmodified geopolymers, such as those containing Cr(VI) [68,70,71]. Further research into optimizing the performance of these geopolymer-based composites, particularly in large-scale applications, will enhance their applicability in a wider range of environmental and industrial contexts.

## Figures and Tables

**Table 1:** Summary of stabilizing agents and mechanisms for heavy metal immobilization in geopolymer matrices.

Raw Materials	Alkali Activator	Stabilizer	Mechanism of Stabilization	Type of Waste	Target Heavy Metals	Ratio Stabilizer/Waste	Leaching Method	Leaching Results	Authors
Metakaolin	NaOH, Na_2_SiO_3_	Na_2_S	Reduction	COPR	Cr (VI)	Molar ratio: from 0 to 10 S^2−^/Cr (VI)	US EPA Method 1311	Without S^2−^, leached Cr (VI) and Cr_tot_ are more than 45 ppm, but with S^2−^ being added with a molar ratio S^2−^/Cr (VI) = 10, leached Cr (VI) and Cr_tot_ are 0.01ppm and 1.37, ppm respectively.	[68]
Metakaolin	NaOH, Na_2_SiO_3_	FeCl_2_·4H_2_O	Reduction	K_2_Cr_2_O_7_	Cr(VI)	Weight ratio 10/30:1	US EPA Method 1311	When the FeCl_2_·4H_2_O addition increased from 0.5 wt.% to 1.5 wt.% with a constant addition (0.1 wt.%) of Cr (VI), the leached Cr_tot_ samples decreased, from 20.05 ppm to 0.42 ppm. When the addition of FeCl_2_·4H_2_O went on increasing from 1.5 wt.% to 3.0 wt.%, leached Cr_tot_ fluctuated slightly, from 0.42 ppm to 0.135 ppm.	[71]
Metakaolin	NaOH, Na_2_SiO_3_	FeSO_4_·7H_2_O	Reduction	K_2_Cr_2_O_7_	Cr(VI), Fe	Molar ratio: Fe(II)/Cr(VI)= 3.5	US EPA Method 1311	For the leachate with 0.1 wt.% of Cr (VI) and without Fe (II) addition, Cr_tot_ and Cr (VI) were 46.9133 ppm and 39.890 ppm. With Fe (II) addition, Cr_tot_ and Cr (VI) leaching values decreased to 0.4576 ppm and 0.057 ppm, respectively. Leached Fe was 0.0469 ppm.	[70]
Metakaolin	NaOH, Na_2_SiO_3_	Na_2_S SGA TBP	Reduction Chelation Chelation	MSWI-FA	Cr, Cu, Cd, Ni, Pb	Weight ratio 1:100	HJ/T300–2007	Leaching values of Cd, Cr, Cu, Ni, and Pb without stabilizers are respectively 0.06, 0.04, 0.015, 0.025, and 0.05 ppm, and they all decreased after adding the chemical stabilizers to <LOQ, 0.01, <LOQ, 0.01, and <LOQ ppm, respectively.	[45]
Fly ash	NaOH, Na_2_SiO_3_	Na_2_S NaH_2_PO_4_ TMT DTC	Reduction Precipitation Chelation Chelation	Cr(NO_3_)_3_,	Cr(III),	Weight ratio 2/3:1	HJ 557-2010	Leached Cr_tot_ without stabilizers containing 3% Cr (NO_3_)_3_ is 1.93 ppm; the best stabilizers are the organic ones (TMT, DTC), at, respectively, 0.59 and 1.05 ppm.	[67]
Fly ash	Na_2_SiO_3_	Na_2_S·9H_2_O	Reduction	Na_2_CrO_4_, PbCrO_4_	Cr(VI)	Weight ratio 1:1	32 × 24 × 22 mm cuboid samples were prepared for leaching tests. The leaching medium volume is 400 mL and the mediums are H_2_O, H_2_SO_4_ (pH = 1), MgSO_4_ 5%, Na_2_CO_3_ 5%.	In deionized water and mineral salts, stabilization increases from less than 20% without stabilizer to more than 99.9% with stabilizer. Improvement in leaching performance in a sulfuric acid medium is nowhere near as great as is observed in any of the other cases, reducing immobilization efficiency to around 80%; this is still much better than the 10% immobilization observed in H_2_SO_4_ without Na_2_S.	[69]
Blast Furnace Slag	NaOH, Water Glass	GSH	Reduction	Chromium-containing sludge	Cr (VI)	Weight ratio 1:200	US EPA Method 1311	GSH pre-reduction doubled CCS incorporation; Cr (VI) leaching reduced significantly. The final product meets landfill and building standards (Cr(VI) < 2.5 mg/L).	[72]

**Table 2:** Summary of stabilizing agents and mechanisms for heavy metal adsorption in geopolymer matrices.

Raw Materials	Activating Solution	Stabilizing Agents	Stabilization Mechanism	Heavy Metals Studied	Adsorption Results	Authors
Metakaolin	NaOH, Na_2_SiO_3_	Sodium Alginate Chitosan	Ion exchange Crystallization reaction	Pb (II)	120.45-142.67 mg/g for Pb (95% removal)	[73]
Metakaolin	NaOH, Na_2_SiO_3_	CTAB	Electrostatic adsorption	Cu (II), Cr(VI)	108.2 mg/g for Cu (92% removal), 95.3 mg/g for Cr(VI) (90%)	[74]
Metakaolin,	NaOH, Na_2_SiO_3_	Sodium Alginate	Ion exchange Physical adsorption	Cu (II)	99% ± 3.4% removal of Cu at pH = 5	[78]
Metakaolin	NaOH	CTAB	Electrostatic attraction Surface complexation	Cu (II)	Complete Cu adsorption at lower concentrations (100% removal)	[77]
Blast Furnace Slag	NaOH, Na_2_SiO_3_	DTC	Chelation	Cd (II)	205.8 mg/g for Cd (static, 85% removal), 382.8 mg/g (dynamic)	[76]

## Data Availability

No new data were created for this work.
